# Long-Term Persisting SARS-CoV-2 RNA and Pathological Findings: Lessons Learnt From a Series of 35 COVID-19 Autopsies

**DOI:** 10.3389/fmed.2022.778489

**Published:** 2022-02-09

**Authors:** Umberto Maccio, Annelies S. Zinkernagel, Reto Schuepbach, Elsbeth Probst-Mueller, Karl Frontzek, Silvio D. Brugger, Daniel Andrea Hofmaenner, Holger Moch, Zsuzsanna Varga

**Affiliations:** ^1^Department of Pathology and Molecular Pathology, University Hospital of Zürich, University of Zurich, Zurich, Switzerland; ^2^Department of Infectious Diseases and Hospital Epidemiology, University Hospital of Zürich, University of Zurich, Zurich, Switzerland; ^3^Institute of Intensive Care, University Hospital Zurich, University Hospital of Zürich, Zurich, Switzerland; ^4^Department of Immunology, University Hospital of Zürich, Zurich, Switzerland; ^5^Institute of Neuropathology, University Hospital Zurich, Zurich, Switzerland

**Keywords:** COVID-19, long-COVID, SARS-CoV-2 RNA PCR, postmortal swabs, pulmonary superinfections, histopathology, autopsy

## Abstract

**Background:**

Long-term sequelae of coronavirus disease 2019 (COVID-19), including the interaction between persisting viral-RNA and specific tissue involvement, pose a challenging issue. In this study, we addressed the chronological correlation (after first clinical diagnosis and postmortem) between severe acute respiratory syndrome coronavirus 2 (SARS-CoV-2) RNA and organ involvement.

**Methods:**

The presence of postmortem SARS-CoV-2 RNA from 35 complete COVID-19 autopsies was correlated with the time interval between the first diagnosis of COVID-19 and death and with its relationship to morphologic findings.

**Results:**

Severe acute respiratory syndrome coronavirus 2 (SARS-CoV-2) RNA can be evident up to 40 days after the first diagnosis and can persist to 94 hours after death. Postmortem SARS-CoV-2 RNA was mostly positive in lungs (70%) and trachea (69%), but all investigated organs were positive with variable frequency. Late-stage tissue damage was evident up to 65 days after initial diagnosis in several organs. Positivity for SARS-CoV-2 RNA in pulmonary swabs correlated with diffuse alveolar damage (*p* = 0.0009). No correlation between positive swabs and other morphologic findings was present. Cerebral (*p* = 0.0003) and systemic hemorrhages (*p* = 0.009), cardiac thrombi (*p* = 0.04), and ischemic events (*p* = 0.03) were more frequent in the first wave, whereas bacterial pneumonia (*p* = 0.03) was more prevalent in the second wave. No differences in biometric data, clinical comorbidities, and other autopsy findings were found.

**Conclusions:**

Our data provide evidence not only of long-term postmortem persisting SARS-CoV-2 RNA but also of tissue damage several weeks after the first diagnosis of SARS-CoV-2 infection. Additional conditions, such as concomitant bacterial pulmonary superinfection, lung aspergillosis, thromboembolic phenomena, and hemorrhages can further worsen tissue damage.

## Introduction

Coronavirus disease 2019 (COVID-19), caused by the beta coronavirus severe acute respiratory syndrome coronavirus 2 (SARS-CoV-2), has been spreading dramatically worldwide since first being reported in Wuhan, China in December 2019 (1).

More than 1 year and a half after the beginning of the pandemic, long-term health consequences of COVID-19 due to persisting SARS-CoV-2 and tissue damage represent an emerging problem, although the pathogenetic mechanisms and the epidemiology of the phenomenon are still largely unknown (2, 3).

COVID-19 can occur with a varying degree of severity (4, 5). Approximately, 33% of patients are asymptomatic (4). Of those who develop symptoms, around 81% experience mild disease, 14% a more severe disease (with respiratory distress), whereas a subset of around 5% of patients progresses to a critical condition (with respiratory insufficiency and/or multi-organ dysfunction) (5). Although the respiratory tract is the most commonly involved organ system (6), patients can also develop cardiovascular complications (7), thromboembolic phenomena (8) including thromboangiitis obliterans (9), several neurologic complications (10), gastroenterological symptoms (11), exuberant inflammatory manifestations (12, 13), and secondary infections (14–17), suggesting that COVID-19 is a systemic disease. The general infection fatality rate is estimated to be 0.68% (18), but it is strongly variable across studies and increases with age and underlying comorbidities (such as arterial hypertension and diabetes) (19, 20).

Long-COVID, in general, used to describe the persistence of symptoms in patients who have recovered from COVID-19, and which cannot be explained by an alternative diagnosis, is thought to occur in up to 10% of cases (21). However, proposals for new classifications aiming to differentiate between acute post-COVID, long post-COVID, and persistent long-COVID are emerging (22).

The pathogenesis of COVID-19 is not fully understood. The angiotensin-converting enzyme 2 (ACE2) and transmembrane protease serine 2 (TMPRSS2) have been shown to be the main receptor and the cofactor for the entry of the virus into the cells (23), but also basigin (CD147) as a receptor (24) and furin as a cofactor (25) play a pivotal role. After the viral attack, complex interplays between humoral and cellular immunity complement activation, cytokines, and coagulation-induced organ damage (26–29). As possible explanations for Long-COVID, several pathogenetic mechanisms, including persistent inflammatory damage, direct viral toxicity in tissues, and post-intensive care syndrome, have been proposed (3, 21).

Autopsies of patients who died from COVID-19 are crucial to gain a better understanding of how SARS-CoV-2 induces damage in human tissues and to consequently improve patient management and therapeutic strategies (30). At the beginning of the pandemic, only few autopsies were performed due to concerns about aerosolization and infectivity of the virus (31). More recently, in compliance with biosafety recommendations of several international regulatory agencies, including the World Health Organization (32), the Centers for Disease Control and Prevention (CDC) (33), and the European Center for Disease Prevention and Control (34), rapidly expanding autopsy literature has become available (35). Nevertheless, although some reports of late histological findings of patients with COVID-19 in the form of single case studies exist, to our knowledge, no autopsy-based studies focusing on the persistence of tissue damage and long-term consequence of SARS-CoV-2 infection have been reported (36).

Additionally, although some clinical studies comparing the first and the second waves of the COVID-19 pandemic exist (37, 38), no detailed autopsy-based studies analyzing the clinical and morphologic differences between the patients who died from COVID-19 in the first compared to the second pandemic wave have been published yet. Moreover, the use of postmortem swabs for detecting the presence of SARS-CoV-2 RNA in autoptic tissues, the postmortem viral distribution, their correlation with the time interval between diagnosis and death, as well as their correlation with morphologic findings have not been extensively studied (39–42).

In view of the foregoing, the aims of our study are: (1) To describe morphologic findings in different organs and tissues and investigate their prevalence. (2) To detect the prevalence of pulmonary superinfection caused by bacteria, viruses, or fungi in patients who died from COVID-19. (3) To analyze possible differences in all those findings between patients who died during the first or second wave of the pandemic. (4) To describe the distribution of SARS-CoV-2 RNA in different organs through postmortem swabs. (5) To correlate the positivity of the postmortem swabs with the time interval between diagnosis of SARS-CoV-2 and death, the incidence of the autoptic morphologic findings with the time interval between diagnosis and death, and the morphologic findings with the positivity of the swabs in the corresponding organs.

## Materials and Methods

### Autopsy Cohort

Overall, 35 autopsies of patients with pre-mortem PCR-confirmed COVID-19 disease were performed at the Department of Pathology and Molecular Pathology of the University Hospital of Zürich, Switzerland.

Seven autopsies (7/35, 20%) were performed during the “first wave” of the COVID-19 pandemic, corresponding to deaths between March 2020 and May 2020. No COVID-19 autopsy was performed between June 2020 and September 2020. The other 28 autopsies (28/35, 80%) were performed during the “second wave” of the COVID-19 pandemic, corresponding to deaths from October 2020 to April 2021. This arbitrary distinction between first and second waves was based on the official classification of the Swiss Federal Office of Public Health and is currently used for comparative purposes by other studies (43, 44).

No cases attributable to any variants of concerns of SARS-CoV-2 according to the WHO definition belonged to this autopsies cohort (45).

Consent to perform the autopsy was given in all cases and the institutional review board (Department of Pathology and Molecular Pathology of the University Hospital Zurich, Switzerland) approved the study. Ethical aspects of research on autopsy tissue of deceased patients, postmortem diagnostics, and molecular analyses were covered in accordance with the Swiss Federal Research Regulations (BASEC Nr. 2020.1316).

### Postmortem Examination and Swabs

All postmortem examinations were conducted in a biosafety Level 3 postmortem facility within an average of 33 hours after death (range, 3–93 hours). After a careful macroscopic examination and photographic documentation, several tissue sites were systematically sampled using a standardized protocol for histological, immunohistochemical, immunofluorescence, and ultrastructural examinations.

Samples for histology and immunohistochemistry were routinely taken from the brain, lungs, heart, liver, spleen, gut, kidney, bone marrow, testicle or ovary, and endocrine organs (pituitary, thyroid, and adrenal glands) and immediately fixed in 4% buffered formalin for 24 hours. Of one patient of the second wave, the brain was not examined according to the declared will of the corresponding relatives.

After paraffin inclusion and microtome sectioning, every histologic sample was processed with conventional stain (hematoxylin and eosin, H&E). Subsequently, the samples were independently examined by two experienced pathologists (U.M. and Z.V.) for major morphological alterations (e.g., inflammation type and distribution, distribution and type of thrombi, infarcts or ischemic changes, signs of superinfection, such as fungal elements or nuclear inclusion suspect of viral infection, major reactive changes, such as metaplasia or hyperplasia of pneumocytes type 2). There was perfect agreement between the two pathologists for every finding in all examined samples (Cohen's kappa coefficient = 1).

During autopsies of the “second wave,” postmortem swabs for SARS-CoV-2-RNA PCR assays from different organs were performed. Postmortem swabs were obtained from a predefined selection of organs: one swab from tracheal secretions, two from the lung parenchyma (one per each lower lobe), one from the myocardium (left ventricle), one from the liver, one from the kidney, one from the small intestine, one from the spleen, and one from the testicles or ovaries. Of one patient, no postmortem swabs were performed, and of another patient only swabs from the lung, heart, and liver were available.

Additional postmortem swabs from the brain (superior frontal gyrus, right) of six patients and from the lamina cribrosa of four patients were available.

During the gross examination, the swabs were taken from each organ after a small sterile incision prior to the dissection of the organ. Access to tracheal fluid was obtained through a small sterile incision of the membranous tracheal part.

Samples were immediately collected in a viral transport medium (cobas® PCR Media, Roche Nr. 06466281190, serving as a nucleic acid stabilizing transport and a storage medium for human specimens) and transported to the Laboratory of Immunology of the University Hospital Zurich, where the presence of SARS-CoV-2 RNA was assessed *via* a real-time reverse PCR assay (cobas® SARS-CoV-2, Roche Nr. 09175431190), a fully automated test for nucleic acid extraction and purification followed by real-time PCR (RT-PCR). Together with the nucleic acid from the sample, the added internal control was simultaneously extracted by adding proteinase and lysis reagent. During the PCR, a sequence of the ORF1 a/b, which is unique to SARS-CoV-2, and a conserved region in the envelope E gene were amplified. The product generated can be measured by detecting the fluorescence. In case of a positive result, the Ct-value (cycle threshold) was indicated (Supplementary Table 1). The Ct-value refers to the number of cycles needed to amplify the viral RNA to reach the predetermined threshold. The lower the Ct value, the more viral RNA was in the sample. The threshold of Ct-value (ORF1 a/b), under which a sample was interpreted as positive, was 40.

In addition, clinical history (including main comorbidities), biometric data [age, gender (male/female)], and body mass index (BMI, in kg/m^2^) of each patient who died during the first and second waves were recorded. Data were obtained from the hospital clinical charts or from the external clinical history submitted to the autopsy (Table 1).

**Table 1:** General characteristic, comparison of the two different cohorts (first vs. second wave) and detailed autopsies' findings.

	**Patients from first wave of pandemic (*n* = 7)**	**Patients from second wave of pandemic (*n* = 28)**	***p*-value**
**Age (years)**	69 (range 45–81)	71 (range 22–89)	0.71
**M/F**	4/7 (57%)	21/28 (75%)	0.38
**BMI (kg/m** ^**2**^ **)**	27.9 (21.6–37.8)	27.4 (17.6–43.6)	0.85
**Main clinical comorbidities**	Cancer (5/7, 71%)	Cancer (9/28, 32%)	0.089
	Arterial hypertension (5/7, 71%)	Arterial hypertension (19/28, 68%)	1.0
	Pulmonary disease (2/7, 28%)	Pulmonary disease (12/28, 43%)	0.68
	Diabetes mellitus (3/7, 43%)	Diabetes mellitus (4/28, 14%)	0.12
	Solid organ transplantation (2/7, 29%)	Solid organ transplantation (2/28, 7%)	0.17
	Bone Marrow transplantation (0/7, 0%)	Bone Marrow transplantation (2/28, 7%)	1.0
	Overweight (3/7, 43%)	Overweight (11/28, 39%)	1.0
	Obesity (2/7, 28%)	Obesity (7/28, 25%)	1.0
**Time interval between diagnosis and**	12 (2–20)	18 (1–65)	0.39
**death (days)**			
**DAD**	6/7 (86%)	18/28 (64%)	0.39
**Bacterial Pneumonia**	2/7 (29%)	21/28 (75%)	**0.03**
**Lung aspergillosis**	0/7 (0%)	6/28 (21%)	0.31
**Viral Pneumonia**	0/7 (0%)	1/28 (4%) (HSV1+CMV)	1.0
**Macroscopic thrombi**	General incidence (3/7, 43%)	General incidence (13/28, 46%)	1.0
	Cardiac ventricle (3/7, 43%)	Cardiac ventricle (2/28, 7%)	**0.04**
	Pulmonary central (0/7, 0%)	Pulmonary central (1/28, 4%)	1.0
	Pulmonary paracentral (0/7, 0%)	Pulmonary paracentral (7/28, 25%)	0.30
	Pulmonary peripheral (0/7, 0%)	Pulmonary peripheral (9/28, 32%)	0.1
	Peripheral veins (0/7, 0%)	Major peripheral vessels (3/28, 11%)	1.0
**Microscopic fibrin thrombi**	General incidence (4/7, 57%)	General incidence (13/28, 46%)	0.69
	Myocardial vessels (1/7, 14%)	Myocardial vessels (2/28, 7%)	0.50
	Pulmonary vessels (2/7, 29%)	Pulmonary vessels (12/28, 43%)	0.68
	Renal (0/7, 0%)	Renal (1/28, 4%)	1.0
	Cerebral vessels (1/7, 14%)	Cerebral vessels (2/28, 7%)	0.5
	Skin (1/7, 14%)	NA	
**Leucocytes thrombi**	General incidence (3/7, 43%)	General incidence (14/28, 50%)	1.0
	Cardiac (1/7, 14%)	Cardiac (4/28, 14%)	1.0
	Pulmonary (3/7, 43%)	Pulmonary (12/28, 43%)	1.0
	Hepatic (1/7, 14%)	Hepatic (0/28, 0%)	0.20
	Mesenterial (1/7, 14%)	Mesenterial (1/28, 4%)	0.36
**Hemorrhages**	General incidence (7/7, 100%)	General incidence (12/28, 43%)	**0.009**
	Brain microhemorrhages (6/7, 86%)	Brain microhemorrhages (3/28, 11%)	**0.0003**
	Subarachnoid (1/7, 14%)	Subarachnoid (2/28, 7%)	0.50
	Subdural hematoma (0/7, 0%)	Subdural hematoma (3/28, 11%)	1.0
	Lung (3/7, 43%)	Lung (5/28, 18%)	0.3
**Infarcts/Ischemia**	General incidence (6/7, 86%)	General incidence (10/28, 36%)	**0.03**
	Cardiac (1/7, 14%)	Cardiac (3/28, 11%)	1.0
	Lung (0/7, 0%)	Lung (5/28, 18%)	0.56
	Small intestine (2/7, 29%)	Small intestine (5/28, 18%)	0.61
	Liver (1/7, 14%)	Liver (8/28, 29%)	0.65
	Cerebral (2/7, 29%)	Cerebral (2/27, 7%)	0.18

*M, male; F, female; DAD, diffuse alveolar damage; BMI, body mass index; HSV1, herpes simplex virus 1; CMV, cytomegalovirus; NA, not available. Bold values are the statistically significant results*.

### Statistical Analyses

Demographic and biometric data from the first and second waves were compared using Student's *t-*test (for age and body mass index, after having found those data to be normally distributed using the Shapiro-Wilk's test, where *p* > α, setting a significance level [α] of 0.05) or Fisher's exact test (for gender, applying this simple 2 x 2 contingency table with one degree of freedom: first vs. second wave/male vs. female) as statistical hypothesis tests.

A Student's *t*-test was also performed to compare the time interval between COVID-19 diagnosis and death between patients who died in the first and second waves (after having found the data to be normally distributed using the Shapiro-Wilk's test, where *p* > α, setting a significance level [α] of 0.05).

Fisher's exact test was also applied to compare the presence of relevant clinical comorbidities (such as cancer history, chronic pulmonary diseases, interstitial lung disease, chronic obstructive pulmonary disorder, pulmonary hypertension, diabetes mellitus, or arterial hypertension) and pathological findings (such as the presence of pulmonary superinfection, cerebral bleedings, etc., as listed in Table 1) between the two waves, applying a simple 2 x 2 contingency table with one degree of freedom (first vs. second wave/the presence of a specific pathologic finding vs. the absence of the same).

Point-Biserial Correlation calculator was applied to compare the positivity of postmortem swabs in each analyzed organ with the time interval between diagnosis and death as well as to compare the incidence of morphologic findings with the time interval between diagnosis and death.

Fisher's exact test was performed to correlate the morphologic findings with the positivity of the swabs in the corresponding organs (positive vs. negative swab/the presence of a specific finding in the organ vs. the absence of the same).

Given the exploratory nature of these analyses, no adjustment for multiple testing was performed.

## Results

### Findings in Patients From the “First Wave”

The average age of the patients was 69 years (range, 45–81 years; standard deviation, 13 years). Four (4/7, 57%) were male, and three (3/7, 43%) were female. The mean time between diagnosis of COVID-19 disease and death was 12 days (range, 2–20 days; standard deviation, 7 days). All patients (7/7, 100%) had one or more chronic comorbidities (arterial hypertension, diabetes mellitus, cancer, overweight, and/or obesity the most common). Two (2/7, 29%) were normal weight (with a BMI between 18.5 and 24.9 kg/m^2^, according to the current WHO classification), three (3/7, 43%) were overweight (BMI between 25.1 and 29.9 kg/m^2^), one (1/7, 14%) was obese Grade I (BMI between 30 and 34.9 kg/m^2^) and one (1/7, 14%) obese Grade II (BMI between 35 and 39.9 kg/m^2^). Average BMI was 27.9 kg/m^2^ (range, 21.6–37.8 kg/m^2^; standard deviation, 5.9 kg/m^2^). Two patients (2/7, 29%, all of whom were male) were solid organ transplant recipients (kidney transplantation 7 and 17 years before death, respectively). One patient was diagnosed with SARS-CoV-2-associated pneumonia 2 months before death, and was successfully treated with conservative therapy and did not require oxygen supplementation, but 1 month after the negativity of SARS-CoV-2 PCR developed a reactivation or reinfection with rapid progression to respiratory insufficiency and death.

Regarding neurological manifestations, one patient had a cerebellar hemorrhage during hospitalization (1/7, 14%), one a severe diffuse brain ischemia 2 days before death (1/7, 14%), and one had recurrent seizures 6 months before death (1/7, 14%). The other four patients (4/7, 57%) had no neurological disorders.

Autopsies were performed on average 33 hours after death (range, 18–56 hours).

The cause of death in six patients (6/7, 86%) was diffuse alveolar damage (DAD), whereas the patient with reactivation/reinfection (1/7, 14%) was found to have massive bacterial pneumonia but no DAD.

Altogether, histopathologic findings consistent with bacterial pneumonia were found in two patients (2/7, 29%), but no fungal or viral pneumonia could be demonstrated.

Three patients (3/7, 43%) had intraventricular macroscopic thrombi. Microscopic fibrin thrombi [in the coronary arteries (1/7, 14%), pulmonary capillaries (2/7, 29%), skin capillaries (1/7, 14%), and cerebral capillaries (1/7, 14%)] as well as leukocytes thrombi [in pulmonary (3/7, 43%), cardiac (1/7, 14%), hepatic (1/7, 14%), and mesenterial capillaries (1/7, 14%)] were also common.

Several patients were also found to have hemorrhages [brain microhemorrhages (6/7, 86%), subarachnoid (1/7, 14%), and pulmonary (3/7, 43%)] and infarcts [small intestine (2/7, 29%) and liver (1/7, 14%)].

Constant adjunctive findings in the lungs were also, to a different degree, endotheliitis of capillaries, alveolar capillary macrophages, prominent hyperplasia of pneumocytes type 2, squamous metaplasia, interstitial edema, lymphocytic and histiocytic inflammation, fibrin-rich alveolar edema, and capillary stasis.

**Figure 1:**
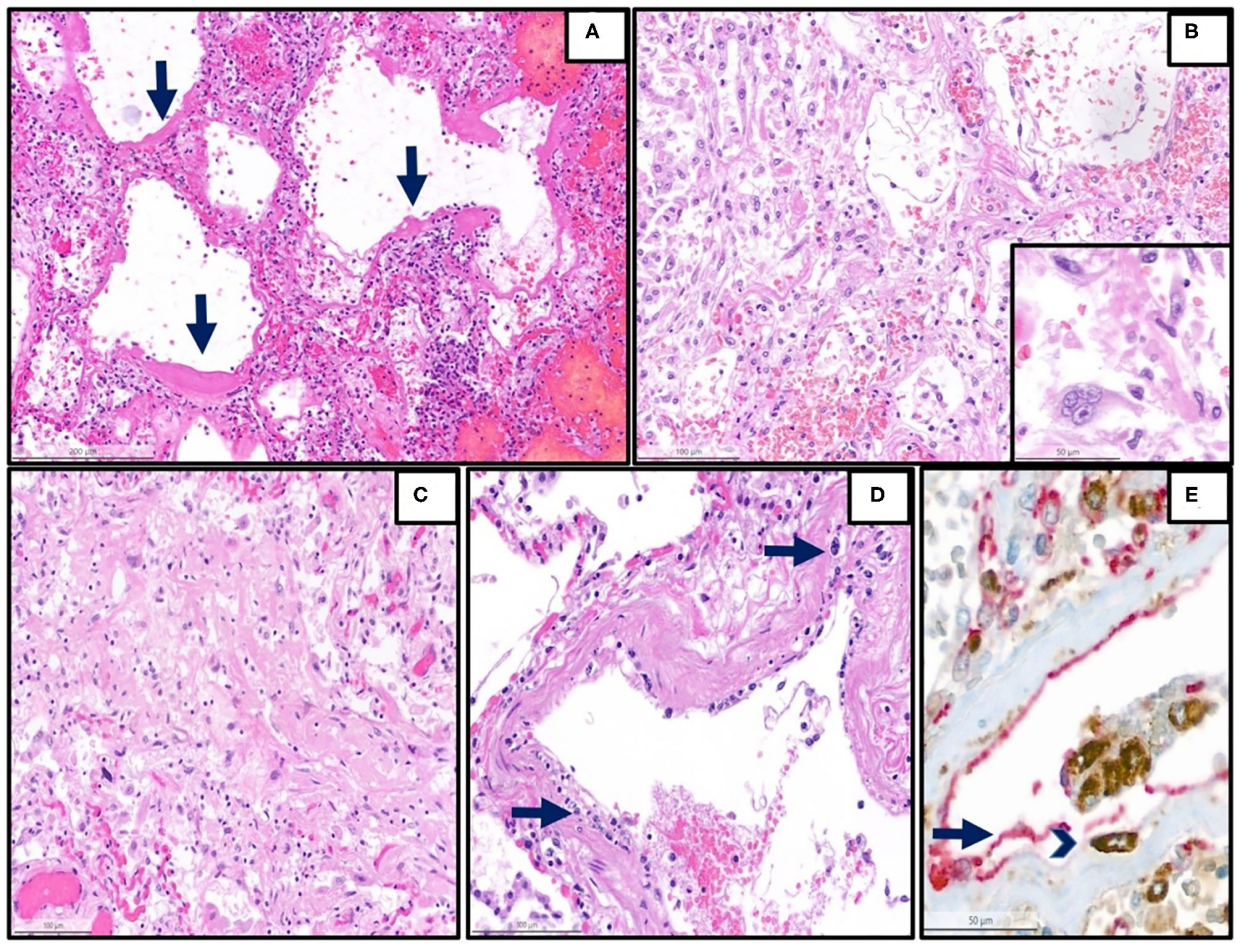
Representative histopathological findings in autopsies from the first wave. **(A)** Lung tissue with diffuse alveolar damage in the exudative phase (hematoxylin and eosin (H&E), magnification 13 x, arrow: hyaline membranes). **(B)** Lung tissue with diffuse alveolar damage in the proliferative phase, which is defined by the presence of organization of the intra-alveolar and interstitial exudate, infiltration with chronic inflammatory cells, and interstitial myofibroblastic reaction. Proliferation and reactive atypias of type II cells are also noted (H&E, magnification 20 x). Inset: reactive pneumocytes type II, magnification 40 x). **(C)** Lung tissue with diffuse alveolar damage in the proliferative phase, showing an excessive collagen deposition (H&E, magnification 20 x). **(D)** Lung arterioles with endotheliitis, which is defined by the presence of subendothelial mononuclear inflammatory infiltrates (arrows) and damage of the endothelium (H&E, magnification 25 x) **(E)** Double immunohistochemistry [red: CD31 (endothelial marker), brown: CD68 (monocytic/macrophage marker)] of another representative case with endotheliitis shows endotheliitis of a venule in the lung with endothelial damage (arrow) and detachment with an associated mononuclear infiltrate (arrowhead) (H&E, magnification 28 x).

**Figure 2:**
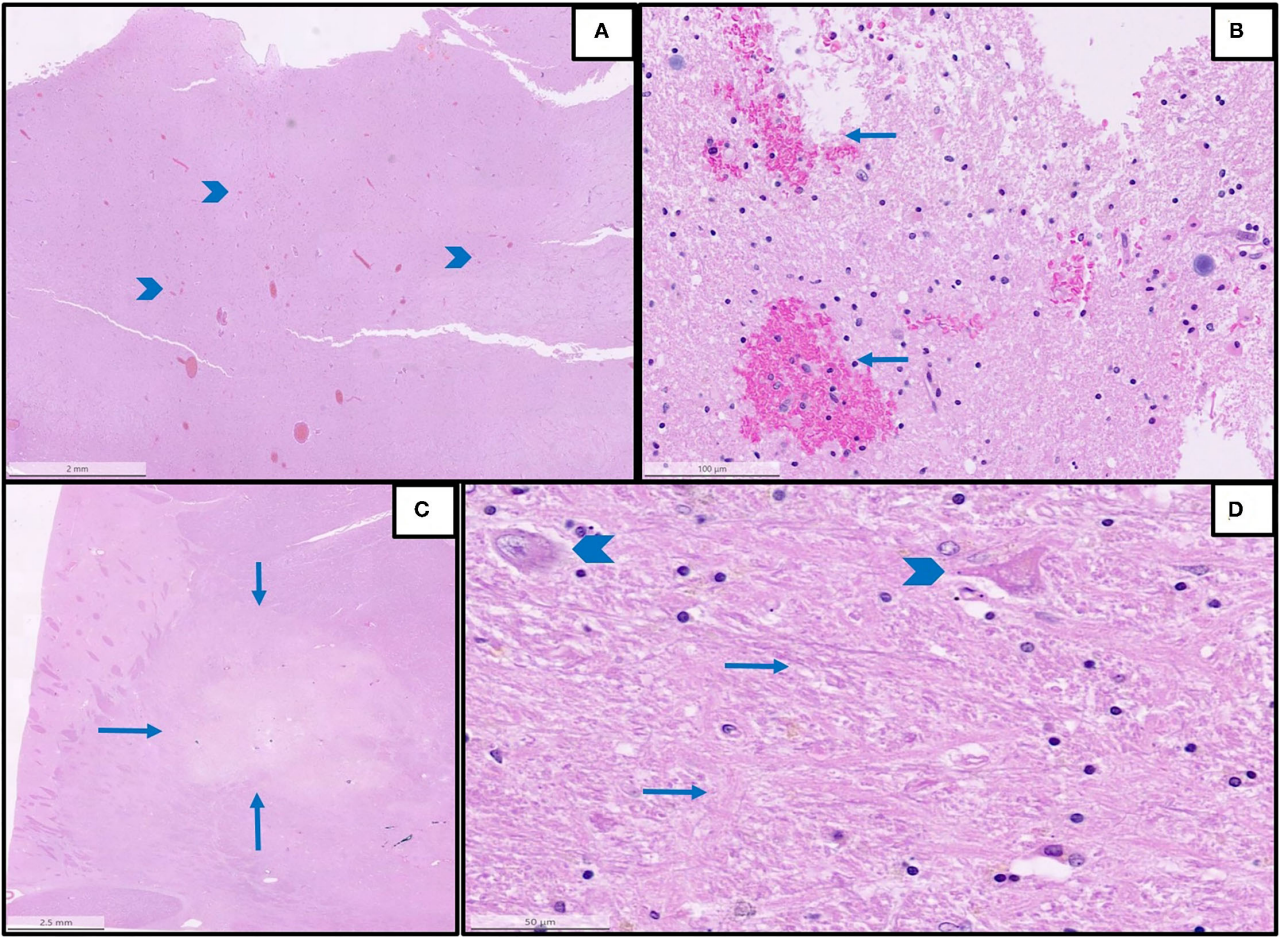
Representative histopathological findings in autopsies from the first wave. **(A)** An overview of multifocal intracerebral microhemorrhages (arrowheads) in a sample from the brain stem (H&E, magnification 1.2 x). **(B)** The detail of image A with microhemorrhages (magnification 25 x, arrows: microhemorrhages). **(C)** An overview of acute brain infarction (arrows) in a sample from basal ganglia (H&E, magnification.62 x). **(D)** Details of image C showing red neurons (arrowheads) and beginning necrosis (arrows) of brain tissue (H&E, magnification 40 x).

Details are summarized in Table 1. Representative images of the most important histopathological findings are shown in Figures 1, 2, and Supplementary Figure 1.

### Findings in Patients From the “Second Wave”

The average age of patients was 71 years (range, 22–89 years; standard deviation, 15 years). Twenty-one (21/28, 75%) were male, and seven (7/28, 25%) were female. The mean time between diagnosis of COVID-19 disease and death was 18 days (range, 1–65 days; standard deviation, 17 days). All patients (28/28, 100%) had one or more severe chronic comorbidities (arterial hypertension, chronic heart failure, cancer, diabetes mellitus, chronic lung disease [COPD (chronic obstructive pulmonary disease) and idiopathic pulmonary fibrosis], autoimmune diseases, chronic kidney disease, asthma, chronic liver disease, and overweight or obesity the most common.

Concerning neurological disorders, four patients had a history of previous ischemic strokes (4/28, 14%), three Alzheimer's disease or unspecified dementia (3/28, 11%), one mild cognitive impairment (1/28, 4%), two multiple cerebral metastases (2/28, 7%), two developed critical-illness neuromyopathy during hospitalization (2/28, 7%), two had a history of previous brain trauma (2/28, 7%), one developed diffuse hypoxic encephalopathy during hospitalization (1/28, 4%), and thirteen had no neurological disorders (13/28, 46%).

Two patients (2/28, 7%) were underweight (BMI <18.5 kg/m^2^, according to current WHO classification), eight (8/28, 29%) normal weight (BMI between 18.5 and 24.9 kg/m^2^), 11 (11/28, 39%) overweight (with a BMI between 25.1 and 29.9 kg/m^2^), three (3/28, 11%) obese Grade I (BMI between 30. and 34.9 kg/m^2^), one (1/28%, 3%) obese Grade II (BMI between 35. and 39.9 kg/m^2^), and three (3/28, 11%) were obese Grade III (BMI > 40 kg/m^2^). Average BMI was 27.4 kg/m^2^ (range, 17.6–43.6 kg/m^2^; standard deviation, 7.2 kg/m^2^).

All patients but one (27/28, 96%) were more than 55 years old at the time of death.

Autopsies were performed on average 33 hours after death (range, 3–93 hours).

Most important, autopsy findings in patients who died in the second wave were diffuse alveolar damage (DAD, 18/28, 64%), macrothrombi (general incidence, 13/28, 46%), microscopic fibrin thrombi (general incidence, 13/28, 46%), and leucocyte thrombi (general incidence, 14/28, 50%), hemorrhages (in particular in cerebral parenchyma, subarachnoid and in the lung, with a general incidence of 12/28, 43%), lung infarcts (5/28, 18%), ischemia of small intestine (5/28, 18%, one among those also with angioinvasive candidiasis of the gut as complication), liver necrosis (8/28, 29%), and cerebral infarcts (2/27, 7%). Similar to patients who died in the first wave, constant adjunctive findings in the lungs were also, to a different degree, endotheliitis of capillaries, alveolar capillary macrophages, prominent hyperplasia of pneumocytes type 2, squamous metaplasia, interstitial edema, lymphocytic and histiocytic inflammation, fibrin-rich alveolar edema, and capillary stasis.

**Figure 3:**
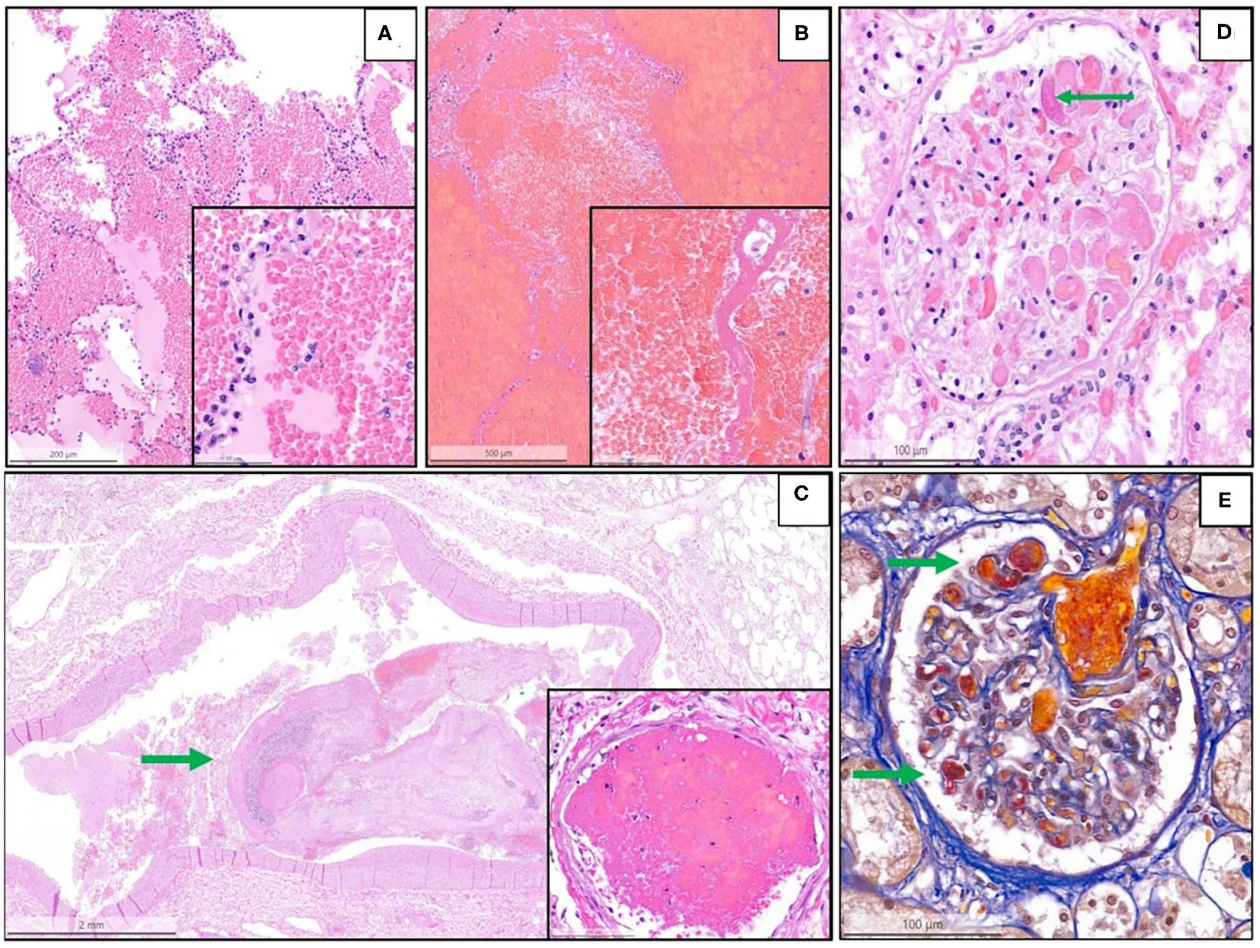
Representative histopathological findings in autopsies from the second wave. **(A)** Acute pulmonary hemorrhage (H&E, magnification 10 x); the inset shows erythrocytes invading alveolar spaces (magnification 40 x). **(B)** Acute hemorrhagic pulmonary infarct (H&E, magnification 5 x); the inset shows a necrotic alveolar septum and hemorrhagic effusion in alveolar space (magnification 40 x). **(C)** Large thrombus (arrow) in a lung arteriole (H&E, magnification 1.4 x); the inset shows a fibrin thrombus in a lung capillary (magnification 28 x). **(D)** Glomerulus (kidney) with fibrin microthrombus (arrow) in a glomerular capillary (H&E, magnification 25 x). **(E)** Acid fuchsin orange G stain (AFOG-stain) shows several fibrin microthrombi (arrows) in the glomerular capillaries (magnification 25 x).

**Figure 4:**
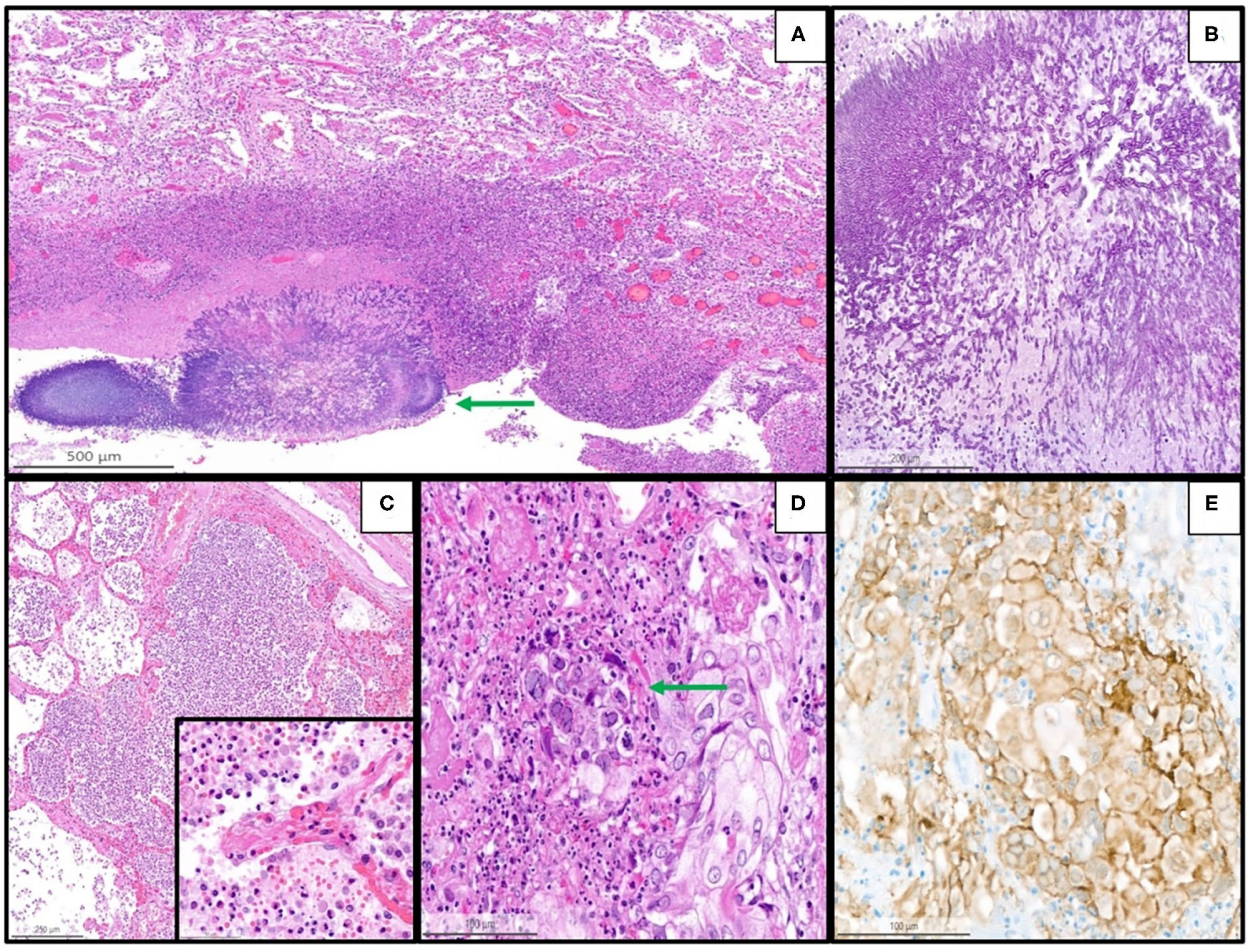
Representative histopathological findings in autopsies from the second wave, illustrating examples of coinfections in the lung of the patients with coronavirus disease 2019 (COVID-19). **(A)** Lung tissue with aspergillosis (arrow) and surrounding acute inflammation (H&E, magnification 10 x). **(B)** Details of image A showing the typical hyphae of Aspergillus *spp*. (PAS, magnification 30 x). **(C)** Acute (bacterial) bronchopneumonia showing granulocytic exudate in the alveolar space and destruction of alveolar septa (H&E, magnification 6 x); the inset illustrates granulocytic inflammation with destruction of a septum (magnification 50 x). **(D)** Herpes simplex pneumonia exhibiting typical herpes-associated nuclear changes (molding, multinucleation, margination of chromatin, see the arrow) (H&E, magnification 25 x). **(E)** Immunohistochemistry for Herpes simplex virus demonstrates a granular cytoplasmic and nuclear positivity along with the typical nuclear changes (magnification 25 x).

**Figure 5:**
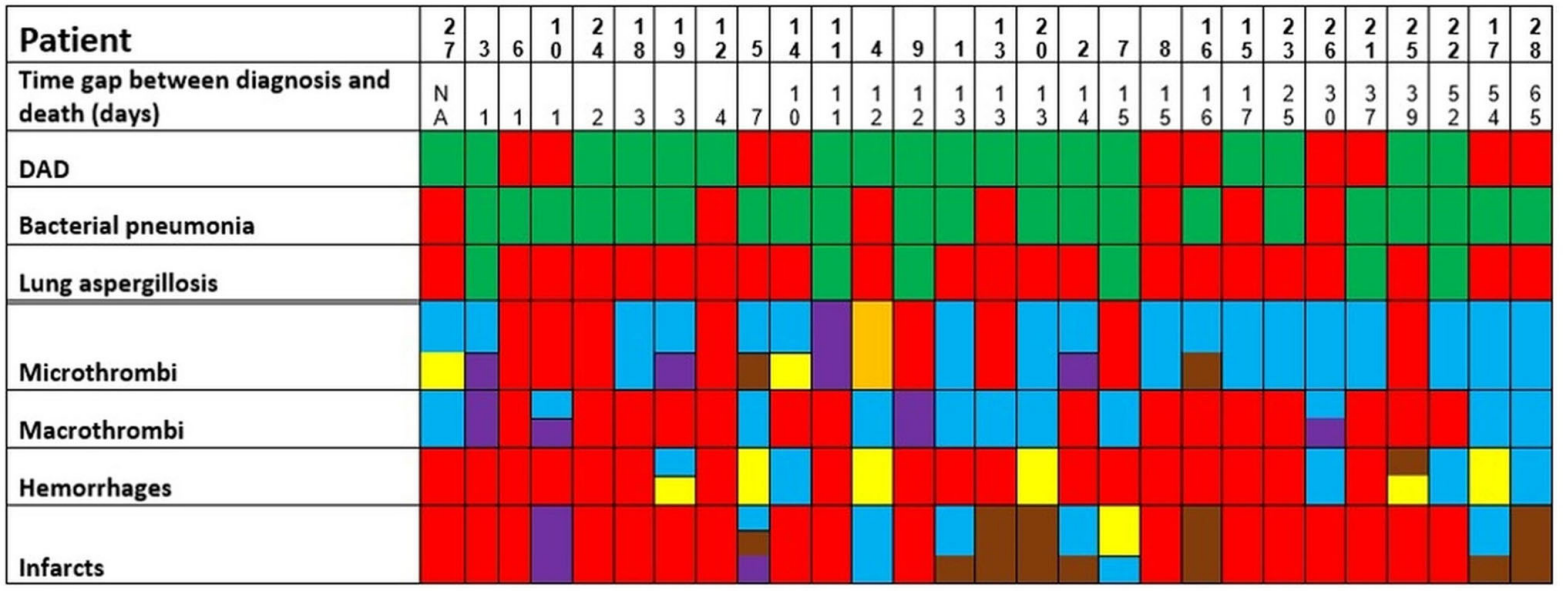
Postmortem findings in the patients of the second wave (green: finding present, red: finding absent. Concerning micro- and macrothrombi, hemorrhages, and infarcts; the following colors to specify the anatomic localization are used: heart and/or major vessels = violet, lung = blue, brain/intracranial = yellow, liver/digestive tract = brown, kidney = orange). The patients are ordered from left to right in a crescent pattern based on the number of days between diagnosis of COVID-19 through nasopharyngeal swab and death. DAD, diffuse alveolar damage; NA, not available.

**Figure 6:**
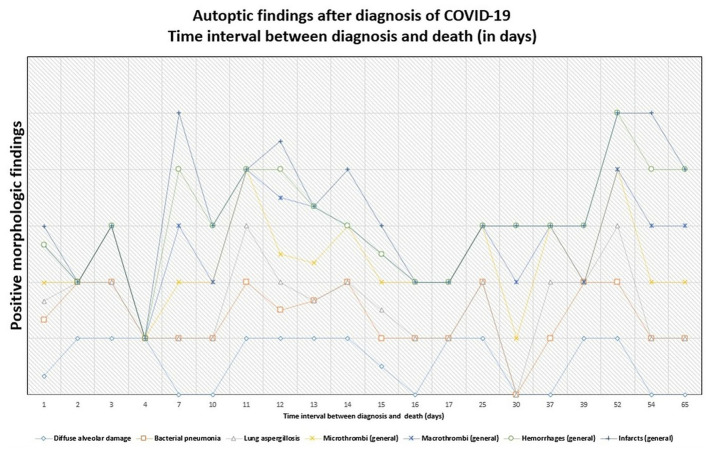
Persistence of histopathologic findings in relation to the time interval between diagnosis and death (in days). The lines do not show absolute percentages but only the relative variation in prevalence of the findings. The different levels of the line along the y-axis are chosen to avoid their overlapping and to facilitate the visual interpretation but do not reflect an absolute percentage, for which we refer the reader to the text.

Details are summarized in Table 1. Representative images of the most important histopathological findings are shown in Figures 3, 4. Additional details of the histopathological findings of this cohort are graphically illustrated in Figures 5, 6.

### Postmortem Swabs for SARS-CoV-2 RNA

Altogether, postmortem swabs were positive for SARS-CoV-2 RNA in the following organs/tissues with the following frequencies: trachea (18/26, 69%), lung (19/27, 70%), heart (8/27, 30%), liver (13/27, 48%), spleen (10/26, 38%), gut (9/26, 35%), kidney (13/26, 50%), testicles (9/19, 47%), ovary (1/7, 14%), brain (2/6, 33%), lamina cribrosa (3/4, 75%). Swabs for SARS-CoV-2 RNA were positive up to 39 days after the first diagnosis of COVID-19 (average, 18 days; range, 1–39 days) and up to 93 hours after deaths (average, 30 hours; range, 3–93 hours).

**Figure 7:**
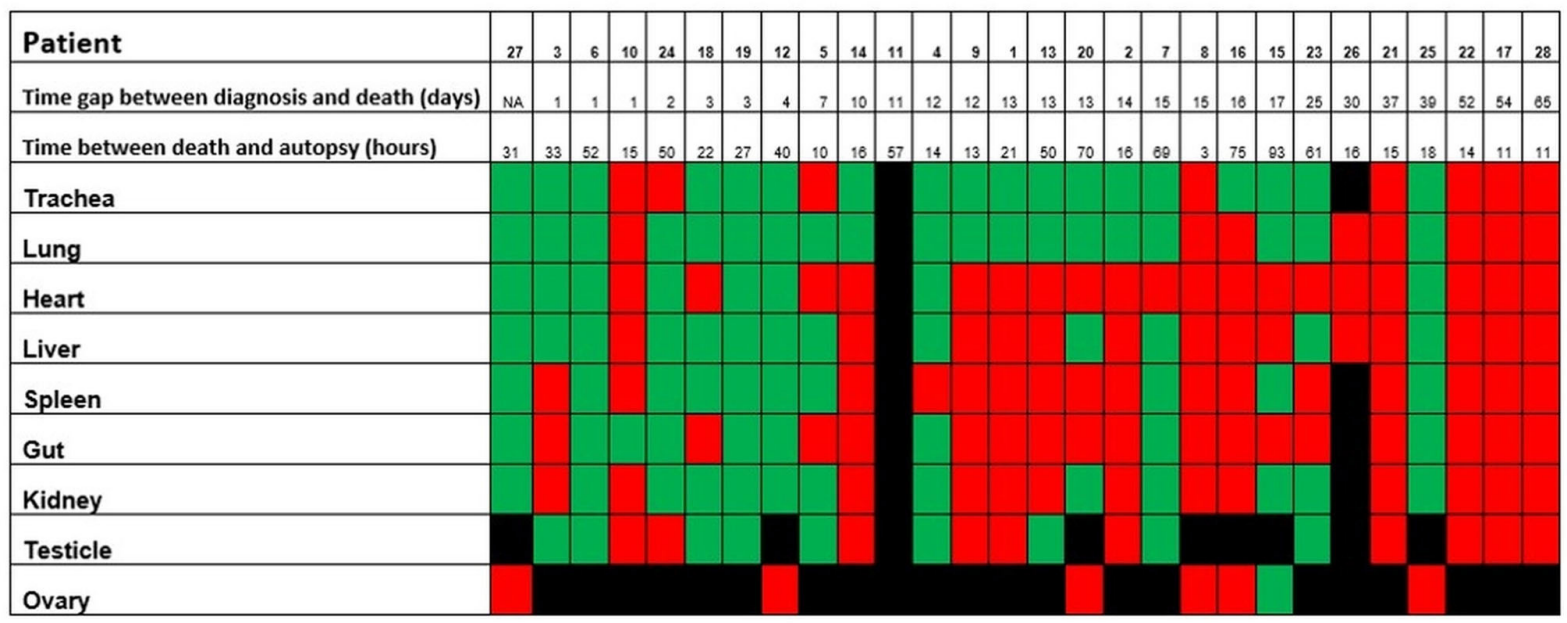
Positivity of the postmortem swabs in the different organs (green: positive, red: negative, black: not available). The patients are ordered from left to right in a crescent pattern based on the number of days between diagnosis of COVID-19 through nasopharyngeal swab and death. Abbreviations: NA, not available.

**Figure 8:**
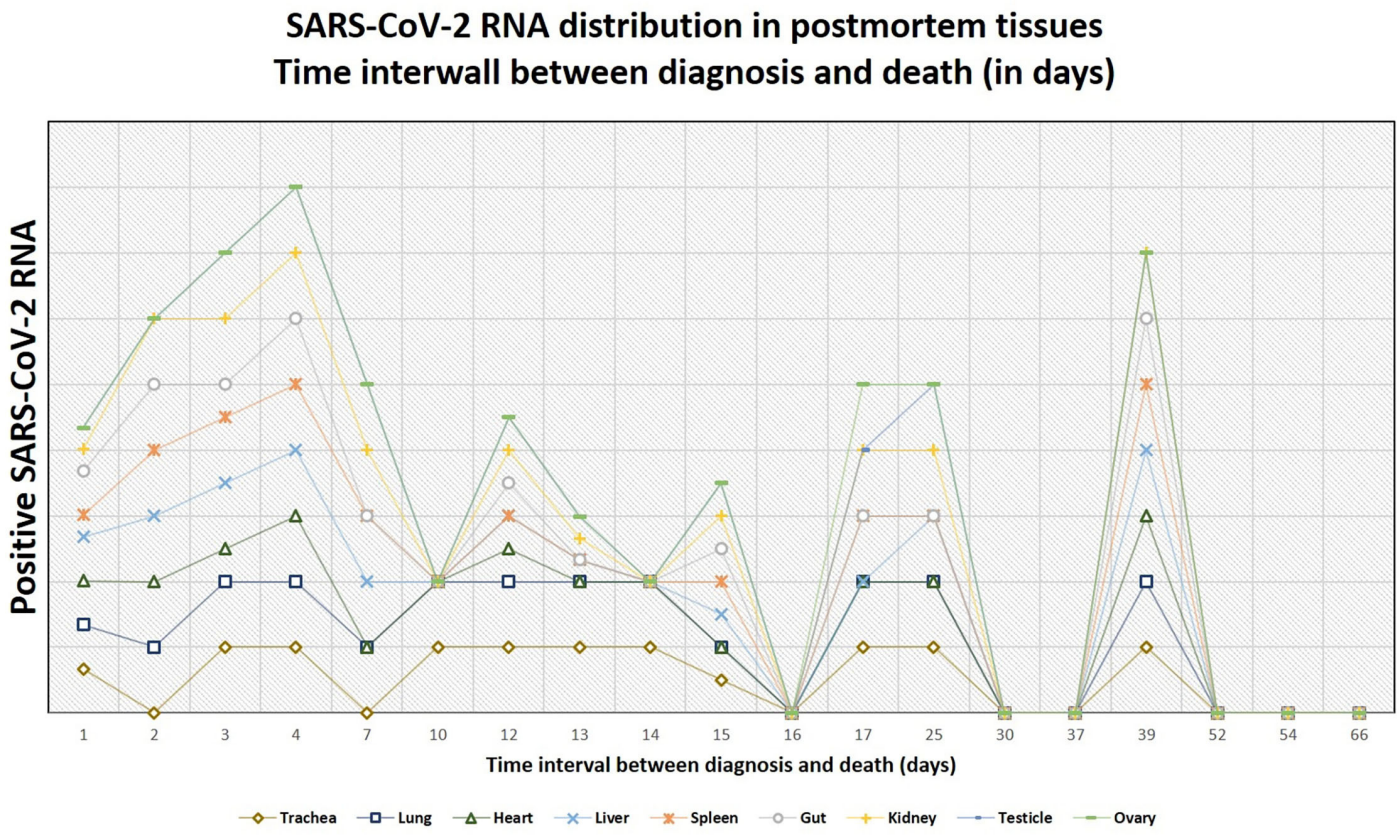
The tendency of positivity of swabs for severe acute respiratory syndrome coronavirus 2 (SARS-CoV-2) RNA in relation to the time interval between diagnosis and death (in days). The lines do not show absolute percentages but only the relative variation in prevalence of the positivity for SARS-CoV-2 RNA. The different levels of the line along the y-axis are selected to avoid their overlapping and to facilitate the visual interpretation but do not reflect an absolute percentage, for which we refer the reader to the text.

**Figure 9:**
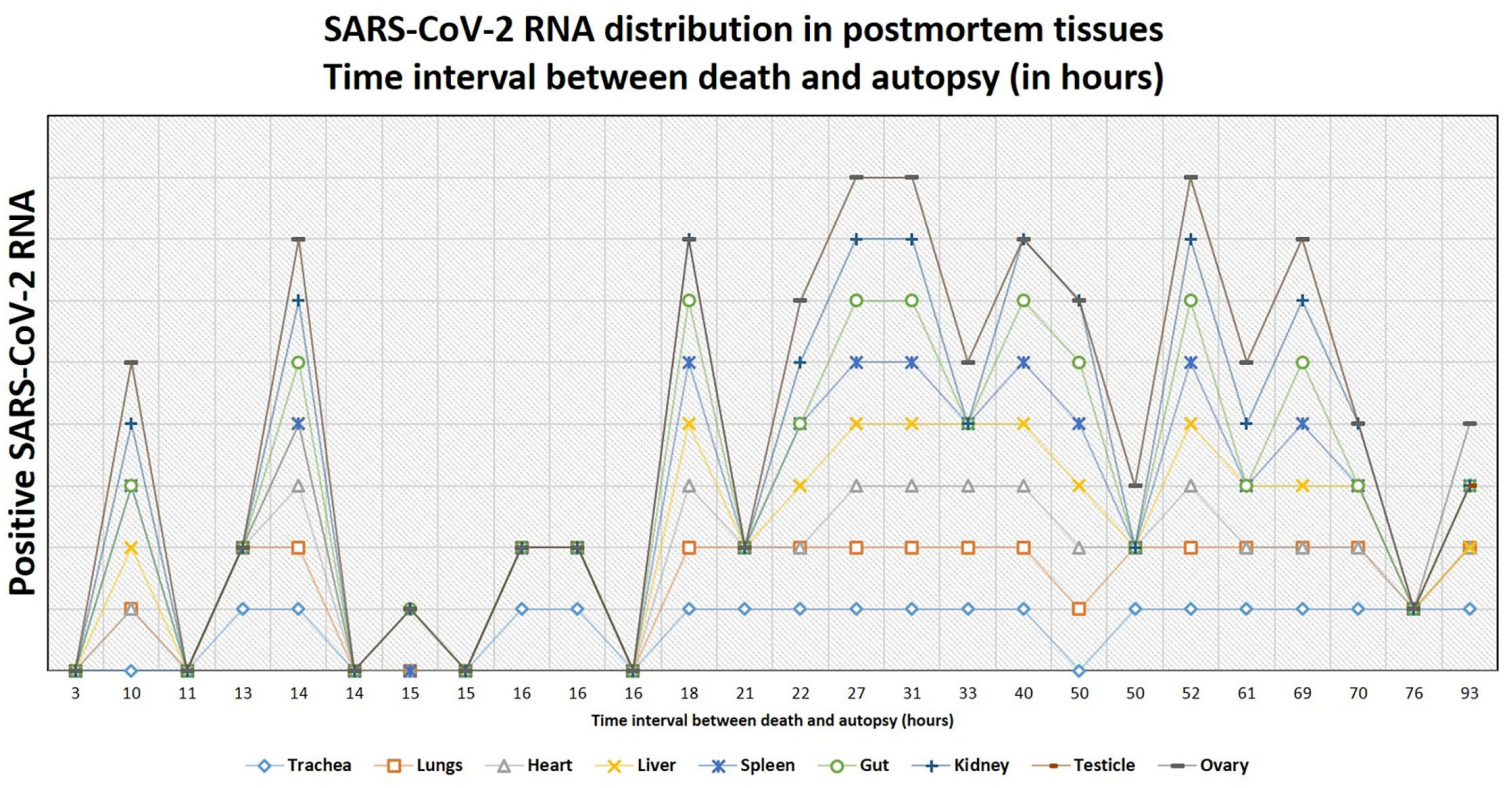
The tendency of postmortem persistence and anatomical distribution of SARS-CoV-2 RNA in relation to the time interval between death and autopsy (postmortem interval, in hours). The lines do not show absolute percentages but only the relative variation in prevalence of the positivity for SARS-CoV-2 RNA. The different levels of the line along the y-axis are chosen to avoid their overlapping and to facilitate the visual interpretation but do not reflect an absolute percentage, for which we refer the reader to the text.

Details are graphically shown in Figures 7–9, and information on Ct-values is provided in Supplementary Table 1.

### Comparison Between the Two Waves

#### Demographic and Biometric Data

No difference concerning age (*p* = 0.71), time interval between diagnosis and death (*p* = 0.39), body mass index (*p* = 0.86), or gender (*p* = 0.38) between patients from the first and second waves could be demonstrated.

#### Clinical Comorbidities

An important comorbidity in patients who died in the first and second waves was a malignancy. Five patients from the first wave (5/7, 71%) and nine from the second wave (9/28, 32%) had an active or treated oncologic disease. Although cancer prevalence was slightly higher in the patients who died in the first than in the patients who died in the second wave, no statistically significant difference between the two groups could be demonstrated (*p* = 0.089).

A history of arterial hypertension was present in five patients of the first wave (5/7, 71%) and in 19 patients of the second wave (19/28, 68%), without statistically significant differences between the patients of the two waves.

Two patients (2/7, 28%) of the first wave had some form of pulmonary disease (COPD and lung hypertension), whereas 12 patients of the second wave (12/28, 43%) had a positive history of pulmonary disease [COPD, ILD (interstitial lung disease), lung hypertension or asthma)], but no difference in the prevalence of lung diseases between the two waves could be demonstrated (*p* = 0.68).

Three patients of the first wave (3/7, 43%) and four of the second wave (4/28, 14%) had diabetes mellitus. Although diabetes was slightly more common in the patients of the first wave, no statistically significant difference in the prevalence of diabetes between the two waves was found (*p* = 0.12).

#### Autopsy Findings

No difference in the time interval between diagnosis of COVID-19 and death could be demonstrated between the patients who died in the first and in the second waves (*p* = 0.39). Moreover, although the finding of DAD was slightly more common in the patients who died in the first wave, no difference between the two cohorts could be identified (*p* = 0.39). Interestingly, the patients of the second wave showed significantly more often (21/28, 75%) bacterial pneumonia (defined histologically by the presence of a granulocytic inflammation with destruction of the lung parenchyma, with or without demonstrable microorganisms through Gram stain) than the patients of the first wave (2/7, 29%) (*p* = 0.03). Another important finding was lung aspergillosis, which was identified in six patients of the second wave (6/28, 21%) but none of the first wave (0/7, 0%), although the difference was not statistically significant (*p* = 0.31).

The general incidence of hemorrhages (independently from the organs considered) was significantly higher in the patients of the first wave (7/7, 100%) than in those of the second wave (14/28, 50%, *p* = 0.009). In more detail, microhemorrhages of the cerebral parenchyma were significantly more frequent among the patients of the first wave (*p* = 0.0003), but no statistically significant difference was observed with regard to hemorrhages in other organs. Similarly, the incidence of ischemic phenomena was significantly higher in the patients of the first wave (6/7, 86%) than in those of the second wave (10/28, 36%) (*p* = 0.03), although no statistically significant differences could be observed if the incidence of ischemic phenomena in the single organs or systems was considered. Intraventricular cardiac thrombi were also more frequent in the patients of the first wave (*p* = 0.04).

**Figure 10:**
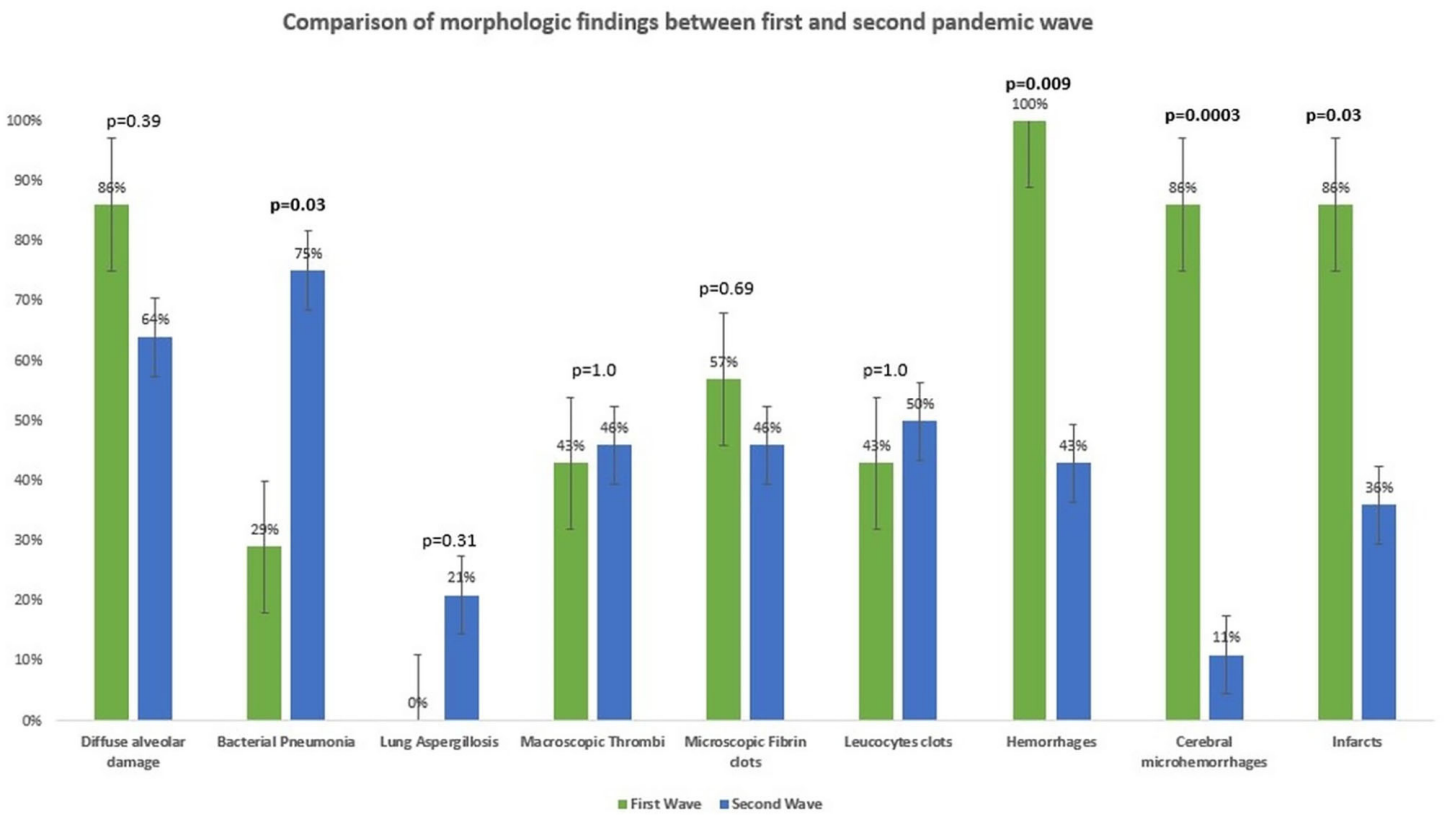
Comparison of morphologic findings at autopsy between first and second pandemic waves.

Details are demonstrated in Table 1 and Figure 10.

### Effect of Time Gap Between Diagnosis and Death

In the point-biserial correlation, a negative *r* between positivity of the postmortem swabs for SARS-CoV-2 RNA and the time interval between diagnosis and death was observed in all examined organs, but only in the lung (*p* = 0.001), trachea (*p* = 0.02), and liver (*p* = 0.03) this association was statistically significant (Supplementary Table 2).

A negative association between the time interval between diagnosis and death and the incidence of DAD and general incidence of infarcts (through a negative *r* in the point biserial correlation) could be demonstrated, although this difference was not statistically significant. In contrast, the association was positive (i.e., with increasing incidence by increasing the time interval between diagnosis and death) for bacterial pneumonia, lung aspergillosis, and general incidence of micro- and macrothrombi, and hemorrhages, but only the association with hemorrhages was statistically significant (*p* = 0.012) (Supplementary Figure 2 and Supplementary Table 3).

### The Effect of Positive Postmortem Swabs in Corresponding Organs

The correlation between morphologic findings and the presence of SARS-CoV-2 RNA in the corresponding postmortem tissues was statistically significant only for diffuse alveolar damage (*p* = 0.0009).

We have additionally divided the cases with a postmortem diagnosis of diffuse alveolar damage in two groups, according to the phase of diffuse alveolar damage (exudative vs. proliferative/organizing phase). No statistically significant association between the phase of diffuse alveolar damage and positivity for SARS-CoV-2 RNA in the lung tissue could be demonstrated (Fisher's exact test, *p* = 0.53). None of those patients had the fibrotic stage of diffuse alveolar damage.

(Figure 5 and Supplementary Tables 4, 5).

## Discussion

Our data provide evidence of postmortem persisting SARS-CoV-2 RNA up to more than 1 month after the first COVID-19 diagnosis. Moreover, tissue damages, observed in this cohort as diffuse alveolar damage, systemic thromboembolic phenomena, ischemic damages, and hemorrhages, could also be demonstrated up to 2 months after the first diagnosis of SARS-CoV-2 infection. Of note, these morphologic findings can last longer than the persistence of SARS-CoV-2 RNA and can be frequently observed without evidence of SARS-CoV-2 RNA in the affected organs. There is a strong association between persistence of SARS-CoV-2 RNA in the lung and diffuse alveolar damage, but not between the persistence of SARS-CoV-2 RNA and other morphologic findings. In cases of diffuse alveolar damage, no statistically significant difference in the positivity of postmortem swabs for SARS-CoV-2 RNA in the lung could be observed between exudative and proliferative phases. Furthermore, we can show that additional conditions, such as concomitant bacterial pneumonia and lung aspergillosis, can occur at any point after the diagnosis of SARS-CoV-2 infection (ranging from very early, i.e., 1 day after diagnosis, to later, i.e., up to 2 months) and can worsen the tissue damage contributing to mortality and morbidity. Those findings together might suggest that tissue damage is not only and always dependent on direct viral effects but could also be mediated through several inflammatory mechanisms and may, therefore, corroborate the hypothesis of possible long-term persistence of COVID-19 in a subset of patients and thus contribute to the understanding of long-COVID disease. Nevertheless, we acknowledge that 35 autopsies represent a limited cohort compared to the total number of fatal cases of COVID-19 in our country, and that an autopsy-based cohort is exposed to too many biases (such as including just the patients with severe comorbidities in a hospital setting) to draw definitive conclusions on the pathogenesis of long-COVID. Moreover, additional studies are needed to explain how many of those morphologic findings (e.g., hemorrhages or infarcts) are directly related to SARS-CoV-2, to different therapies, or to the clinical settings of critically ill patients, as well as to better characterize harms and benefits of therapies.

This study shows that the most important autoptic findings in the patients who died from COVID-19 were diffuse alveolar damage (24/35, 69%) and concomitant bacterial pneumonia (23/35, 66%). Lung aspergillosis was also a relevant superinfection in many patients of the second wave (6/28, 21%), known as an important cause of death in critically ill patients. In general, the most important cause of death was diffuse alveolar damage, and, according to the interpretation provided in the autopsy report, SARS-CoV-2 was seen as the major responsible factor in death in most cases (26/35, 74%). In all other patients but one, bacterial pneumonia with or without concomitant lung aspergillosis was the main cause of death, and SARS-CoV-2 was a relevant comorbidity (8/35, 23%). Only one patient (1/35, 3%), aged 22, died from the complications of a myocardial infarction, and the concomitant SARS-CoV-2 infection did not contribute to mortality.

The biometric data, prevalence of clinical comorbidities, and morphologic findings of this autopsy-based cohort are in line with those reported in a recently published review by *Caramaschi et al*. on 58 studies on autopsies or biopsies of overall 662 patients with COVID-19, providing further confirmation of the spectrum and characteristics of this new disease (46).

To the best of our knowledge, no previous studies compared biometric and clinical data as well as autoptic findings between the first and second pandemic waves. Of note, only the general incidence of hemorrhages and of brain microhemorrhages was significantly higher among the patients of the first wave compared to those of the second wave (respectively, *p* = 0.009 and *p* = 0.0003). On this point, brain microhemorrhages in COVID-19 are well-known radiologic and autoptic findings (47–51) and are significantly more frequent in the patients with severe disease and in the ICU (intensive care unit) setting (52). According to the literature, their general incidence ranges from 6.9–54% (52), but, to our knowledge, no studies compare the incidence of cerebral microhemorrhages between the two waves. Nevertheless, caution is needed while interpreting these results; the first point to consider is a sampling bias, given the fact that the protocol for brain sampling was not as standardized as for other organs and the quantity of samples varies among autopsies.

Moreover, autopsy findings do not necessarily reflect the situation of critically ill patients, considering that an autoptic examination is performed only in a minor percentage of cases. However, the present result remains interesting and further confirms that COVID-19 is a systemic disease. Cerebral microhemorrhages are indeed a well-known complication in patients with severe COVID-19 and maybe associated with cerebral endotheliitis (35, 53), although they could also be observed in critically ill patients without COVID-19 in the context of critical illness-associated microbleeds (CRAM) (54). Again, those considerations are of utmost importance when evaluating the harms and benefits of different therapies in severe COVID-19.

The association between severe COVID-19 and bacterial superinfection as well as lung aspergillosis has already been reported in the literature, although the incidence varies among studies (55–57). In our cohort, the incidence of bacterial pneumonia was significantly higher in the second wave. Moreover, the patients of the second wave developed lung aspergillosis more frequently, but this difference was not statistically significant. Among others, a possible explication for these differences is the use of dexamethasone as standard therapy in severe COVID-19 during the second pandemic wave. Given the small number of patients of this cohort, only speculations are possible, and larger studies are necessary to validate this hypothesis.

Apart from that, no statistically significant difference between the patients of the first and of the second pandemic wave concerning age, gender prevalence, body mass index, time interval between diagnosis of SARS-CoV-2 and death, prevalence of main comorbidities, and of most autoptic findings was demonstrated. However, due to the small sample size of the entire cohort, no substantial differences between the first and second waves can be drawn and additional studies, in particular, metanalyses might be necessary to better understand the epidemiologic differences between the pandemic waves.

In patients of the second wave, postmortem swabs were most positive in the lung (70%) and the trachea (69%). SARS-CoV-2 RNA could be detected up to 93 hours after death. The persistence of SARS-CoV-2 RNA several hours and even days after death is a described phenomenon in several organs (58–60), and has been detected up to 35 days after death according to one case report (61). A possible limitation in the interpretation of these results might be that positive SARS-CoV-2 RNA does not necessarily reflect the presence of the virus in the tissues. Actually, false-positive results are reported to occur in about 2% of cases in living patients (62), being contamination during collection procedures, extraction or amplification (e.g., through aerosolization in containment hood), or cross-reaction with other viruses (e.g., other Coronaviruses), some of the factors which might contribute to false-positive RT-PCR results (63). Postmortem RT-PCR for SARS-CoV-2 in tissues of the upper respiratory tract has shown a sensitivity of 96.8% and a specificity ranging from 94.2 to 97.5% (64), so that false-positive and false-negative results should be taken into account. Even a true positive result does not always reflect a viable virus. A surrogate estimating virus viability may be the ct-value of the RT-PCR test: a study by *Jaafar et al*. showed that the culture of the virus is successful up to a ct-value of 25 (70%), but at a ct-value of 30, this value drops to 20%, and values above 35 are associated with only a low likelihood of successful culture (3%) (65). In virtue of these considerations, additional studies to assess virus viability from autopsy tissues are needed.

Of note, in the second wave, typical histological signatures of COVID-19, such as endotheliitis, leucocytic- or fibrin-thrombi, could be demonstrated also in organs where no SARS-CoV-2 RNA was detected through postmortem swabs and in the patients in whom SARS-CoV-2 was not the main cause of death. Moreover, in our cohort, apart from diffuse alveolar damage, no significant correlation was found between swabs' positivity and morphologic findings. These findings further support the role of indirect and persisting viral damage rather than a direct viral effect of SARS-CoV-2 (27, 65, 66).

This result partially agrees with an autopsy-based study of *Skok et al*., where no correlation between viral load and severity of organ damages at autopsy was found (41).

In our cohort, there was an inverse association between the time elapsed from diagnosis and death and the positivity of swabs in the organs (i.e., the more time passes, the less frequent positivity is observed), but this was statistically significant only for the lung, the trachea, and the liver. Despite this tendency, we found positive postmortem swabs in different organs also in the patients who were diagnosed with COVID-19 several days before, and one patient had positive swabs of trachea, lung, heart, gut, liver, and kidney even 39 days after the diagnosis. Importantly, we found a statistically significant correlation between SARS-CoV-2 RNA evidence in swabs in the lungs and the occurrence of diffuse alveolar damage (*p* = 0.0009), but the association between other morphologic findings and positivity of the swabs in the corresponding organs was not statistically significant.

Up to date, postmortem viral tropism and dynamics through swab examinations have not been characterized in detail. Two autopsy-based studies of *Skok et al*. investigated the presence of SARS-CoV-2 RNA on different organs at autopsy and found a systemic distribution of the virus (in the throat, lung, intestine, and brain) and morphologic findings in line with ours (41, 42).

Another autopsy-based study of *Deinhardt-Emmer et al*., including 11 patients who died from COVID-19, described the viral distribution through postmortem swabs performed in a relatively short time interval after death (mean, 5.6 hours; range, 1.5–15 hours). The authors demonstrated a systemic involvement with the highest viral load in the lungs and hypothesized a topological correlation of viral load and histopathological damage in the lung (39).

In general, our results on postmortem swabs and morphologic findings are in line with the aforementioned autopsy-based studies, providing further confirmation of the systemic nature of COVID-19 and of the tropism of SARS-CoV-2 for different organs. Moreover, the lack of correlation between the presence of SARS-CoV-2 RNA in tissues and morphologic findings seems again to confirm an inflammatory-mediated process, rather than direct viral damage, as already postulated in the literature (66–70).

In summary, SARS-CoV-2 RNA persists in different organs several weeks after the first diagnosis of COVID-19. Macroscopic and histologic pieces of evidence of tissue damage in critically ill patients can be demonstrated up to 2 months thereafter both in organs with and without postmortem evidence of SARS-CoV-2 RNA. Cerebral microhemorrhages were statistically more frequent in the patients of the first pandemic wave. Postmortem SARS-CoV-2 RNA showed a systemic distribution, with the highest prevalence in the lungs and trachea. There is a significant correlation between the presence of SARS-CoV-2 RNA and diffuse alveolar damage in the lungs, but no other correlation with other autoptic findings.

## Data Availability Statement

The original contributions presented in the study are included in the article/Supplementary Material, further inquiries can be directed to the corresponding author.

## Ethics Statement

The studies involving human participants were reviewed and approved by Swiss Ethics. The patients/participants provided their written informed consent to participate in this study.

## Author Contributions

UM designed the study, collected samples, analyzed morphologic data, performed statistical analyses, interpreted clinical and pathological data, and drafted the article. AZ designed the study, interpreted clinical and pathological data, and drafted the article. RS designed the study, interpreted clinical and pathological data, and drafted the article. EP-M performed PCR analyses on postmortem swabs, interpreted clinical and pathological data, and drafted the article. KF, SB, and DH interpreted clinical and pathological data and drafted the article. HM designed the study, analyzed morphologic data, interpreted clinical and pathological data, and drafted the article. ZV designed the study, analyzed morphologic data, interpreted clinical and pathological data, drafted the article, and coordinated the cooperation among the authors. All the authors were involved in critical reading, writing the article, and had final approval of the submitted and published version.

## Funding

The experiments were covered by the Internal University Research Funds of the Department of Pathology and Molecular Pathology, University Hospital Zurich Switzerland.

## Conflict of Interest

The authors declare that the research was conducted in the absence of any commercial or financial relationships that could be construed as a potential conflict of interest.

## Publisher's Note

All claims expressed in this article are solely those of the authors and do not necessarily represent those of their affiliated organizations, or those of the publisher, the editors and the reviewers. Any product that may be evaluated in this article, or claim that may be made by its manufacturer, is not guaranteed or endorsed by the publisher.

## Supplementary Material

Supplementary Figure 1 Details of Figures 2C,D (cerebral tissue from basal ganglia). Inset 1: Luxol-stain demonstrates myelin loss (magnification 25 x). Inset 2: immunostaining for NF70/200 (magnification 25 x) demonstrates loss of axons: as such, myelin loss should be interpreted as secondary to infarction and not as demyelination. Inset 3: Luxol-stain in a control case from the same anatomical region (basal ganglia) without myelin loss. Inset 4: immunostaining for NF70/200 in a control case from the same anatomical region (basal ganglia) without loss of axons.

Supplementary Figure 2 Comparison between morphologic findings and positivity of postmortem swabs in the corresponding organs. The patients are ordered from up to down in a crescent pattern based on the number of days between diagnosis of COVID-19 through nasopharyngeal swab and death. Interpretation: green = finding present with positive swab; blue = finding present with negative swab; red = finding absent with positive swab; black = finding absent with negative swab. NA, not available; DAD, diffuse alveolar damage; BP, bacterial pneumonia; LA, lung aspergillosis; MiT, microthrombi; MaT, Macrothrombi; LH, lung hemorrhages; LI, lung infarcts; MI, myocardial infarction.
