# Supplementary figures and images for: Long-Term Persisting SARS-CoV-2 RNA and Pathological Findings: Lessons Learnt From a Series of 35 COVID-19 Autopsies
Source: Front Med (Lausanne). 2022 Feb 9;9:778489. doi: 10.3389/fmed.2022.778489 (PMC8865372; doi:10.3389/fmed.2022.778489)

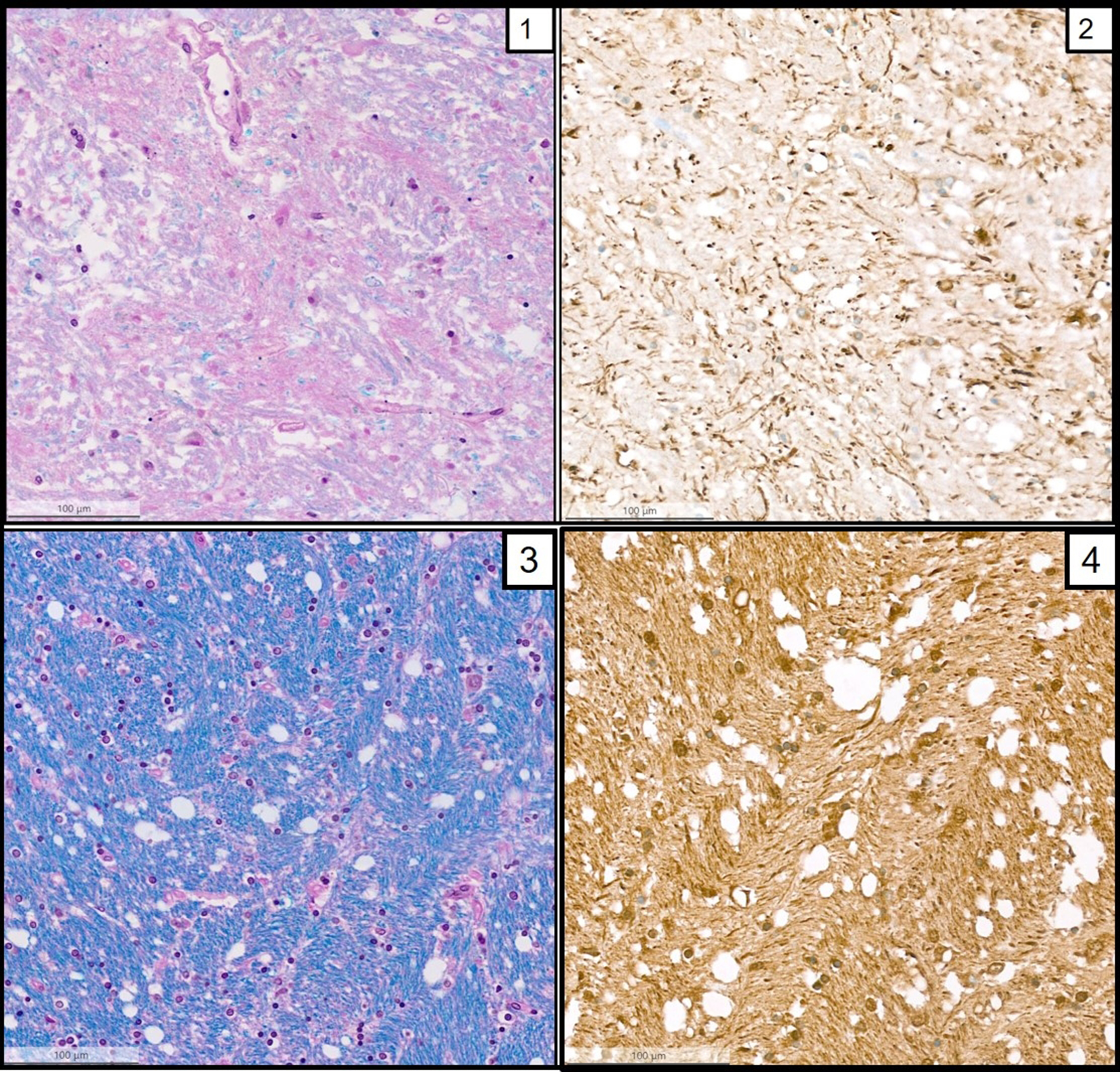

Supplement: Supplementary Figure 1 — Details of Figures 2C,D (cerebral tissue from basal ganglia). Inset 1: Luxol-stain demonstrates myelin loss (magnification 25 x). Inset 2: immunostaining for NF70/200 (magnification 25 x) demonstrates loss of axons: as such, myelin loss should be interpreted as secondary to infarction and not as demyelination. Inset 3: Luxol-stain in a control case from the same anatomical region (basal ganglia) without myelin loss. Inset 4: immunostaining for NF70/200 in a control case from the same anatomical region (basal ganglia) without loss of axons. [file Image_1.jpg]

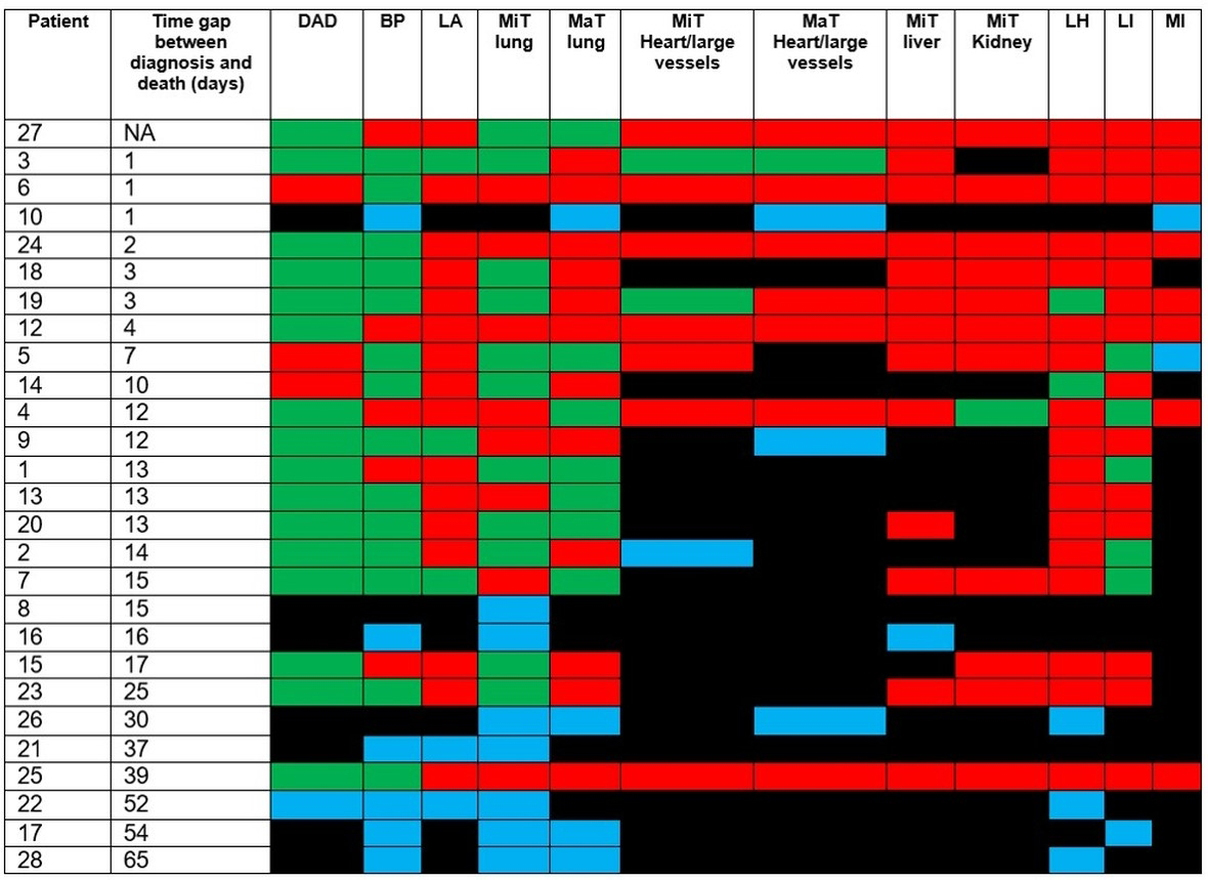

Supplement: Supplementary Figure 2 — Comparison between morphologic findings and positivity of postmortem swabs in the corresponding organs. The patients are ordered from up to down in a crescent pattern based on the number of days between diagnosis of COVID-19 through nasopharyngeal swab and death. Interpretation: green = finding present with positive swab; blue = finding present with negative swab; red = finding absent with positive swab; black = finding absent with negative swab. NA, not available; DAD, diffuse alveolar damage; BP, bacterial pneumonia; LA, lung aspergillosis; MiT, microthrombi; MaT, Macrothrombi; LH, lung hemorrhages; LI, lung infarcts; MI, myocardial infarction. [file Image_2.jpg]
