# Supplementary material for: Long-Term Persisting SARS-CoV-2 RNA and Pathological Findings: Lessons Learnt From a Series of 35 COVID-19 Autopsies
Source: Front Med (Lausanne). 2022 Feb 9;9:778489. doi: 10.3389/fmed.2022.778489 (PMC8865372; doi:10.3389/fmed.2022.778489)
Supplement: Supplementary file 3 [file Table_1.docx]

**Supplementary materials**

| **Patient** | **Time interval between diagnosis and death**  **(days)** | **Ct-value**  **Trachea** | **Ct-value**  **Lung** | **Ct-value**  **Heart** | **Ct-value**  **Liver** | **Ct-value**  **Gut** | **Ct-value**  **Spleen** | **Ct-value**  **Kidney** | **Ct-value**  **Testicle** | **Ct-value**  **Ovary** | **Ct-value Brain** | **Ct-value lamina cribrosa** |
| --- | --- | --- | --- | --- | --- | --- | --- | --- | --- | --- | --- | --- |
| **1** | **13** | **38.75** | **38.11** | **-** | **-** | **-** | **NA** | **-** | **NA** | **NA** | **NA** | **NA** |
| **2** | **14** | **25.86** | **29.05** | **-** | **-** | **-** | **NA** | **-** | **NA** | **NA** | **-** | **NA** |
| **3** | **1** | **17.74** | **20.23** | **31.9** | **25.9** | **-** | **NA** | **-** | **-** | **NA** | **NA** | **13.54** |
| **4** | **12** | **24.95** | **27.1** | **33.97** | **27.56** | **21.99** | **-** | **32.34** | **32.92** | **NA** | **NA** | **14.61** |
| **5** | **7** | **NA** | **25.27** | **-** | **27.56** | **16.9** | **-** | **30.99** | **26.67** | **NA** | **NA** | **NA** |
| **6** | **1** | **16.44** | **15.24** | **31.8** | **27.88** | **27.59** | **NA** | **25.95** | **25.4** | **NA** | **NA** | **NA** |
| **7** | **15** | **30.2** | **29.73** | **-** | **33.04** | **34.81** | **36.58** | **32.87** | **33.77** | **NA** | **NA** | **NA** |
| **8** | **15** | **-** | **-** | **-** | **-** | **-** | **-** | **-** | **NA** | **-** | **NA** | **NA** |
| **9** | **12** | **32.95** | **33.79** | **-** | **-** | **-** | **-** | **-** | **-** | **NA** | **NA** | **NA** |
| **10** | **1** | **-** | **-** | **-** | **-** | **-** | **-** | **-** | **-** | **NA** | **NA** | **31.61** |
| **11** | **11** | **NA** | **NA** | **NA** | **NA** | **NA** | **NA** | **NA** | **NA** | **NA** | **NA** | **NA** |
| **12** | **4** | **20.82** | **18.1** | **30.63** | **29.5** | **31.35** | **29.74** | **30.79** | **NA** | **NA** | **NA** | **NA** |
| **13** | **13** | **28.14** | **29.79** | **-** | **-** | **NA** | **-** | **-** | **32.36** | **NA** | **NA** | **NA** |
| **14** | **10** | **21.82** | **23.16** | **-** | **-** | **-** | **-** | **-** | **-** | **NA** | **NA** | **NA** |
| **15** | **17** | **29.9** | **30.53** | **-** | **-** | **-** | **31.39** | **31.12** | **NA** | **33.5** | **NA** | **NA** |
| **16** | **16** | **32.86** | **-** | **-** | **-** | **-** | **-** | **-** | **NA** | **NA** | **-** | **NA** |
| **17** | **54** | **-** | **-** | **-** | **-** | **-** | **-** | **-** | **-** | **NA** | **-** | **NA** |
| **18** | **3** | **21.35** | **29.72** | **-** | **30.59** | **-** | **32.09** | **32.14** | **33.29** | **NA** | **-** | **NA** |
| **19** | **3** | **18.18** | **19.58** | **26.74** | **26.48** | **18.91** | **29.21** | **25.49** | **29.32** | **NA** | **27.51** | **NA** |
| **20** | **13** | **31.92** | **32.61** | **-** | **NA** | **NA** | **NA** | **NA** | **NA** | **NA** | **NA** | **NA** |
| **21** | **37** | **-** | **-** | **-** | **-** | **-** | **NA** | **-** | **-** | **NA** | **NA** | **-** |
| **22** | **52** | **-** | **-** | **-** | **-** | **-** | **-** | **-** | **-** | **NA** | **NA** | **NA** |
| **23** | **25** | **22.29** | **16.47** | **-** | **26.82** | **-** | **-** | **24.02** | **NA** | **NA** | **NA** | **NA** |
| **24** | **2** | **-** | **25.74** | **31.53** | **30.9** | **31.16** | **NA** | **28.32** | **-** | **NA** | **34.03** | **NA** |
| **25** | **39** | **29.81** | **NA** | **NA** | **NA** | **NA** | **NA** | **NA** | **NA** | **-** | **NA** | **NA** |
| **26** | **30** | **NA** | **NA** | **NA** | **NA** | **NA** | **NA** | **NA** | **NA** | **NA** | **NA** | **NA** |
| **27** | **NA** | **21.95** | **27.8** | **27.09** | **NA** | **NA** | **NA** | **25.78** | **NA** | **-** | **NA** | **NA** |
| **28** | **65** | **-** | **-** | **-** | **-** | **-** | **-** | **-** | **-** | **NA** | **NA** | **NA** |

**Supplementary Table 1.** Ct-values (ORF1 a/b). Legend: “NA”: not available/not applicable (either the value was evaluated as positive but the value is not available anymore in the electronic system at the time of data analysis or the PCR was not performed). “-“: negative result (> 40 cycles).

|  | **Point-biserial correlation (r)** | **p-value** |
| --- | --- | --- |
| **Trachea** | -0.43 | **0.02** |
| **Lung** | -0.59 | **0.001** |
| **Heart** | -0.32 | 0.09 |
| **Liver** | -0.42 | **0.03** |
| **Spleen** | -0.32 | 0.1 |
| **Gut** | -0.32 | 0.1 |
| **Kidney** | -0.33 | 0.1 |
| **Testicle** | -0.41 | 0.09 |
| **Ovary** | -0.1 | 0.8 |

**Supplementary Table 2.** Comparison between positivity of the postmortem swab and the time interval between diagnosis and death in days (Point-biserial correlation).

|  | **Point biserial correlation (r)** | **p-value** |
| --- | --- | --- |
| **DAD** | -0.038 | 0.85 |
| **Bacterial pneumonia** | 0.088 | 0.66 |
| **Lung aspergillosis** | 0.11 | 0.60 |
| **Microthrombi** | 0.27 | 0.17 |
| **Macrothrombi** | 0.085 | 0.66 |
| **Hemorrhages** | 0.47 | **0.012** |
| **Infarcts** | -0.025 | 0.9 |

**Supplementary Table 3.** Comparison between morphologic findings and the time interval between diagnosis and death (Point-biserial correlation).

| **Morphologic findings** | **p-value** |
| --- | --- |
| Diffuse alveolar damage | **0.0009** |
| Bacterial Pneumonia | 1.0 |
| Lung aspergillosis | 0.65 |
| Pulmonary microthrombi | 0.20 |
| Pulmonary macrothrombi | 0.68 |
| Lung hemorrhage | 0.14 |
| Lung infarcts | 0.63 |

**Supplementary Table 4.** Correlation between morphologic findings and positivity of postmortem swabs in the corresponding organs (exact Fisher’s test).

|  | **Postmortem swabs for SARS-CoV-2-RNA** | **Positive** | **Negative** |
| --- | --- | --- | --- |
| **Phase of DAD** | **Exudative** | 6 | 0 |
|  | **Proliferative/organizing** | 10 | 2 |

**Supplementary Table 5.** Differentiation between exudative and proliferative/organizing phases of diffuse alveolar damage (DAD) and their relationship with positivity with postmortem swabs for SARS-CoV-2 RNA in the lung. There is no statistically significant difference between positivity and the phase of DAD (p = .53).
